# Flexible Passive Sensor Patch with Contactless Readout for Measurement of Human Body Temperature

**DOI:** 10.3390/bios13060572

**Published:** 2023-05-23

**Authors:** Marco Zini, Marco Baù, Alessandro Nastro, Marco Ferrari, Vittorio Ferrari

**Affiliations:** 1 Department of Information Engineering, University of Brescia, 25123 Brescia, Italy; 2 INO-CNR (National Institute of Optics—National Research Council), Via Branze 45, 25123 Brescia, Italy

**Keywords:** body temperature sensor, coil-coupled sensor, passive sensor, RLC resonator, contactless, time-gated technique, autocorrelation

## Abstract

A passive flexible patch for human skin temperature measurement based on contact sensing and contactless interrogation is presented. The patch acts as an RLC resonant circuit embedding an inductive copper coil for magnetic coupling, a ceramic capacitor as the temperature-sensing element and an additional series inductor. The temperature affects the capacitance of the sensor and consequently the resonant frequency of the RLC circuit. Thanks to the additional inductor, the dependency of the resonant frequency from the bending of the patch has been reduced. Considering a curvature radius of the patch of up to 73 mm, the maximum relative variation in the resonant frequency has been reduced from 812 ppm to 7.5 ppm. The sensor has been contactlessly interrogated by a time-gated technique through an external readout coil electromagnetically coupled to the patch coil. The proposed system has been experimentally tested within the range of 32–46 °C, giving a sensitivity of −619.8 Hz/°C and a resolution of 0.06 °C.

## 1. Introduction

Monitoring body temperature is crucial to diagnose the state of health and determine flaws related to human diseases in advance [1]. Typically, human body temperature is measured by relying on either contact or noncontact approaches. Contact approaches are typically more reliable, but may require the use of disinfecting agents and longer measurement time [2]. Contactless approaches provide faster temperature measurements at the expense of a lower reliability due to the interface medium transmission coefficient and sensor distance from the skin [2,3]. However, when continuous body-temperature measurements are required, e.g., in domestic or hospital environments, light, unobtrusive and battery-less contact sensors would be typically demanded. To achieve this purpose, the exploitation of flexible wearable sensors is progressively emerging as an effective approach for monitoring human body temperature [4,5,6,7]. Near-Field Communication (NFC)-based temperature sensors have been successfully employed in biomedical applications to develop battery-less skin patches [8]. These sensors employ on-board active electronic integrated circuits, typically a microcontroller with one or more connected sensors, that have to be energized by a reader to transmit the information retrieved through a digital communication protocol. As an alternative, solutions based on passive resonant sensors that can be suitably employed with flexible wearable sensors have been demonstrated [9,10].

A typical drawback of flexible sensors is the possible dependency of their performance on the bending of the substrate housing the sensing element [11,12], requiring appropriate techniques to eliminate or mitigate this undesired effect [13].

In this paper, a novel approach is proposed for human skin-temperature measurement that is based on a passive flexible patch combining contact-sensing with contactless interrogation. Specifically, this work extends this technique to a flexible sensor patch attached onto a curved surface and achieves contactless interrogation independently of the curvature radius of the flexible patch.

The fabricated patch can be stuck on the skin, e.g., the arm of a subject, and eventually be covered by clothing since a contactless interrogation technique is performed from a nearby interrogation unit.

Contactless measurement techniques have been adopted as an effective solution to interrogate sensors in applications where cabled or battery powered approaches are not feasible or are invasive [14,15].

Specifically, passive resonant sensors and electromagnetic interrogation techniques have been successfully validated for quartz crystal resonators (QCR) [16], resonant piezo-layer (RPL) sensors [17], MEMS resonators [18] and LC resonant sensors [15,19]. Both frequency-domain and time-domain approaches can be used for the contactless interrogation of such sensor type [16]. Frequency-domain techniques measure impedance, reflection coefficients or a specific transfer function by simultaneously exciting and sensing the resonator [20,21,22]. Conversely, time-domain techniques, as proposed in this paper, exploit the transient free response of the resonator by separating the excitation and detection phases in time [23]. For both techniques, the independency of the sensing quantities from the reading distance has been demonstrated [16].

The solution proposed in this work exploits a contactless interrogation technique based on the magnetic coupling between a passive sensor unit (SU) and an electronic interrogation unit (IU). The SU is based on an RLC resonant circuit and embeds a flexible patch with an inductive copper coil for magnetic coupling, a ceramic capacitor as the temperature-sensing element and an additional inductor to make the resonant frequency of the SU independent of the unavoidable bending of the patch that is inherent to the body conformation and movements. The IU includes a primary coil with front-end electronics for contactless excitation and read-out. The proposed approach of interrogating the passive SU offers high accuracy and, at the same time, paves the way to the fabrication of a fully integrated compact system, since the use of bench instrumentation for the readout of the resonant frequency is not required [24,25].

## 2. System Description

**Figure 1:**
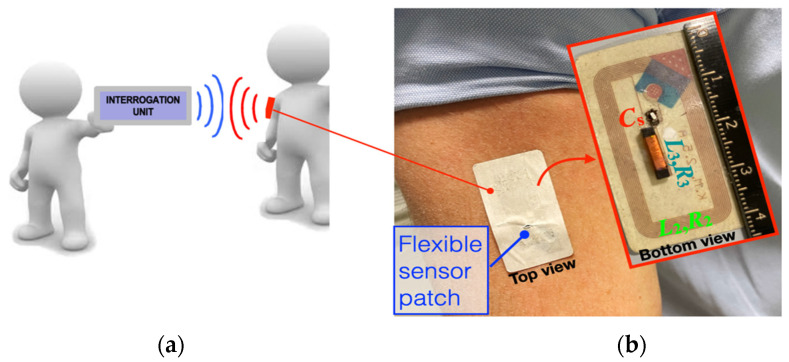
(**a**) Typical application scenario for the temperature sensor patch; (**b**) top and bottom view of the sensor patch stuck on the arm skin of a human subject.

Figure 1a shows the typical application scenario for the proposed flexible patch, while Figure 1b reports the top and bottom views of the proof-of-concept prototype patch stuck on the arm skin of a human subject, respectively. The proposed sensor is expected to be positioned on body parts, e.g., the arm, neck or temple, with a curvature radius typically in the order of 70 mm. The patch is composed of a planar sensor coil with 10 turns of traces patterned onto a copper foil on an adhesive paper substrate of 43 mm × 23 mm.

The planar coil, with resistance *R*_2_ and inductance *L*_2_ is connected in series to the inductor with parameters *R*_3_ and *L*_3_, and to the ceramic capacitor *C*_s_, which was adopted as the sensing element based on its temperature coefficient of capacitance (TCC). Both *R*_3_ and *C*_s_ are commercially available, lightweight components with millimeter-size dimensions, chosen not to limit the flexibility of the patch. An appropriate performance and adequate comfort can be obtained by adjusting the size and form factors of the components, as well as their placement with respect to the expected main bending direction of the patch. The capacitor *C*_s_ is coated with an epoxy resin, which prevents capacitance variation due to humidity. The contact between *C*_s_ and the skin required to measure temperature demands additional passivation of the soldering joints to avoid degraded sensing performances due to sweat or moisture.

**Figure 2:**
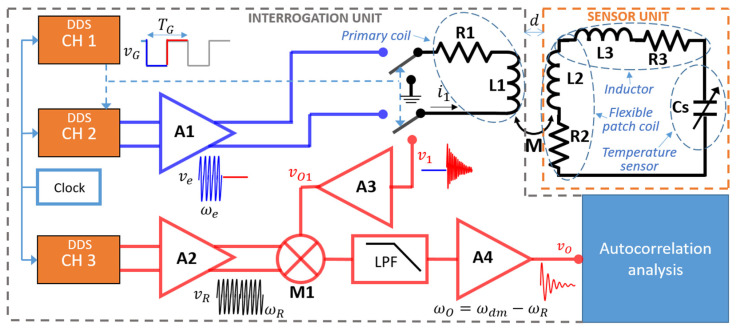
Block diagram of the proposed contactless interrogation system.

The patch represents the SU of the proposed contactless interrogation system and its equivalent RLC circuit is shown in Figure 2, along with a schematic diagram of the IU. The primary coil *L*_1_ of the IU is magnetically coupled with the coil *L*_2_ of the SU through the mutual inductance *M*, which depends on the distance *d* between the coils.

The IU generates, by means of a Direct Digital Synthesis (DDS) chip with three independent channels, namely CH1, CH2 and CH3, the sinusoidal signals used to excite the SU and demodulate the signal received from the SU.

The excitation and detection phases are timed from the gating signal *v*_G_(*t*) generated by CH1 with period *T*_G_. In the excitation phase, a sinusoidal signal *v*_e_(*t*) at angular frequency *ω*_e_ driving the coil *L*_1_ is generated by CH2 and amplified by A1.

Choosing *ω*_e_, close to *ω*_s_ = 2*πf*_s_, the current induced in the coil *L*_2_, through the magnetic coupling with *L*_1_, causes the RLC circuit to resonate at its natural resonant frequency *f*_s_ with quality factor *Q*, given by:

(1) $$f_{s}=\frac{1}{2\pi \sqrt{\left(L_{2}+L_{3}\right)C_{s}}},$$

(2) $$Q=\frac{1}{R_{2}+R_{3}}\sqrt{\frac{L_{2}+L_{3}}{C_{s}}}.$$

Among the adopted components, the planar coil is the element that mostly depends on the bending or deformations of the patch. However, assuming *L*_3_ >> *L*_2_ and *R*_3_ >> *R*_2_, both *f*_s_ and *Q* are independent of the electrical parameters of the planar coil, thus making the bending uninfluential.

After the excitation phase ends, the excitation signal *v*_e_(*t*) is switched off and the detection phase begins. The resonator undergoes free decaying oscillations at an angular frequency *ω*_dm_ *= ω*_s_(1 − 1/(4*Q*^2^))^1/2^, inducing a current in *L*_2_. For *Q* ≈ 100, this results in a relative deviation (ω_s_ – ω_dm_)/ω_s_ ≈ 10^−5^. Consequently, the voltage *v*_1_(*t*) across *L*_1_ can be sensed and amplified by A3, providing the voltage *v*_O1_(*t*), which is down-converted by the analog multiplier M1 that mixes it with the reference signal *v*_R_(*t*) = *V*_R_*cos*(*ω*_R_*t*) generated by CH3 and amplified by A2.

The resulting signal is fed to the low-pass filter LPF and amplified by A4, leading to the sinusoidal damped signal *v*_O_(*t*) with angular frequency *ω*_O_ = |*ω*_dm_ − *ω*_R_| and decay time *τ*_m_ = 2*Q*/*ω*_s_. As validated in [23], *ω_O_* and *τ*_m_ are not affected by the interrogation distance *d*.

To determine *ω*_O_, the signal *v*_O_(*t*) is digitized and its autocorrelation function *R*_xx_(*τ*) is computed. The expression of *R*_xx_(*τ*) is:

(3) $$R_{\mathrm{xx}}\left(\tau \right)=\frac{1}{4}{\left(MA_{O}\right)}^{2}e^{-\left|\tau \right|/\tau_{m}}\left[\tau_{m}cos\left(\omega_{o}\tau \right)+\frac{cos\left(\omega_{o}\tau +2\theta_{m}+\arctan\left(\omega_{o}\tau_{m}\right)\right)}{\sqrt{1/\tau_{m}^{2}+\omega_{o}^{2}}}\right].$$

The autocorrelation function *R*_xx_(*τ*) can be used to determine *ω*_O_. Assuming that the reference frequency *ω*_R_ is constant and stable, the original angular frequency of *v*_O1_(*t*) can be derived as *ω*_dm_ = *ω*_R_ ± *ω*_O_, and its variation Δ*ω*_dm_ can be attributed to the frequency variations in the SU resonant RLC circuit.

## 3. Experimental Results

The fabricated SU was experimentally characterized by means of an impedance analyzer (HP4194A), obtaining a resonant frequency *f*_s_ at room temperature of 1.634 MHz. The SU coil has *R*_2_ = 5.53 Ω and *L*_2_ = 5.07 µH without bending, while the additional series inductor with dimensions of 10 mm × 2.5 mm has *R*_3_ = 196.85 Ω and *L*_3_ = 545.25 µH, measured at *f*_s_. The capacitive temperature-sensing element is a ceramic capacitor with *C*_s_ = 17.02 pF at 20 °C. The IU coil, which is a 6-turn planar coil milled from an 80 mm × 80 mm standard flame-retardant (FR4) substrate, has *R*_1_ = 7.18 Ω and *L*_1_ = 8.94 μH.

**Figure 3:**
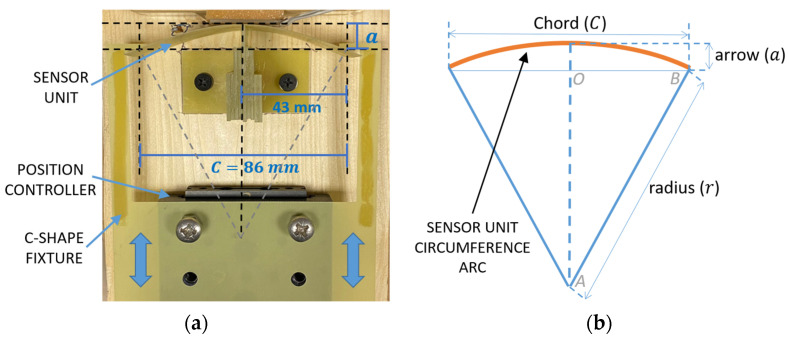
(**a**) Developed setup composed of a position controller with micrometric resolution that allows to bent the flexible support for the SU patch to test the effect on the resonant frequency; (**b**) schematic representation of the flexible support circumference arc.

Firstly, the effects of different patch bending conditions on the sensor coil were investigated by measuring the corresponding variations in *L*_2_ and *R*_2_. Figure 3a shows the dedicated setup, which was purposely adopted to ensure a controlled bending of the patch. The patch was attached to a flexible FR4 support. A C-shaped fixture with an aperture *C* = 86 mm was installed on a micrometric position controller, which allows for the flexible support to be grabbed close to its outer edges.

The micrometric position controller forces a displacement *a* of the outer edges, and thus a bending of the patch. With reference to Figure 3b, assuming that the deformation can be approximated to an arc of a circumference, by applying Pythagorean theorem to the triangle AOB the curvature radius *r* can be derived as:

(4) $$r=\frac{C^{2}}{8a}+\frac{a}{2}.$$

**Figure 4:**
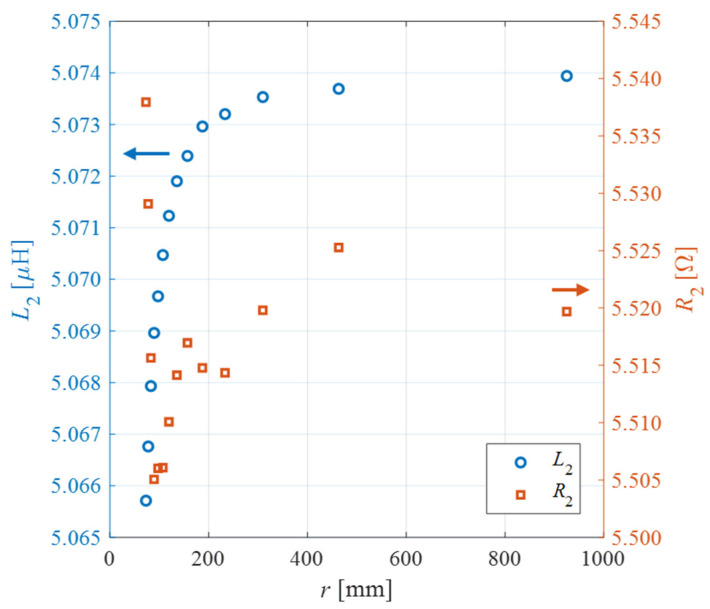
Measurement results of the inductance *L*_2_ and resistance *R*_2_ of the SU coil as function of the curvature radius *r*.

Figure 4 shows the values of *L*_2_ and *R*_2_ as function of *r* from 73 to 925 mm, measured at the SU resonant frequency *f*_s_ = 1.634 MHz. As expected, the inductance of the patch monotonically rises for increasing curvature radius values [11,12,13]. According to (1), the variation of *L*_2_ induces a variation in *f*_s_. Considering the case with *L*_3_ = 0, the maximum relative variation in *f*_s_ caused by bending with respect to *f_s_*_0_ when *a* = 0 results in Δ*f_s_*/*f_s_*_0_ = 812 ppm. On the other hand, when *L*_3_ is placed in series with *L*_2_, the maximum relative variation of *f*_s_ decreases to Δ*f_s_*/*f_s_*_0_ = 7.5 ppm. In summary, the insertion of the additional inductance advantageously reduces the frequency relative variation due to bending by two orders of magnitude.

According to (1) and (2), the theoretical values of *f*_s_ and *Q* with the additional inductor *L*_3_ and *a* = 0 are *f*_s0_ = 1.644 MHz and *Q*_0_ = 28.

**Figure 5:**
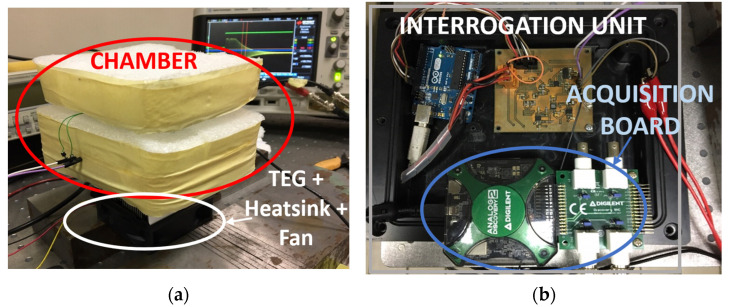
(**a**) Plastic box test chamber surrounded by polystyrene foam for thermal insulation equipped with a Peltier thermoelectric element at the bottom to set the temperature of the inner volume; (**b**) interrogation unit and acquisition board used to test the proposed system.

To test the sensor ability to detect the temperature *T*, a tailored experimental setup was fabricated, as shown in Figure 5a. A chamber with an internal volume of (80 × 80 × 40) mm^3^ was assembled from a plastic box surrounded by polystyrene foam for thermal insulation.

A Peltier thermoelectric element installed at the bottom of the chamber was used to electrically set the temperature of the inner volume.

The sensor patch was placed on the top of the chamber in adherence with the cover. A Pt1000 temperature sensor read by a multimeter (Fluke 8840) was placed in thermal contact with the SU and used as a reference temperature sensor. The primary coil *L*_1_ of the IU was placed outside the cover at a distance *d* = 2 mm from the SU patch.

**Figure 6:**
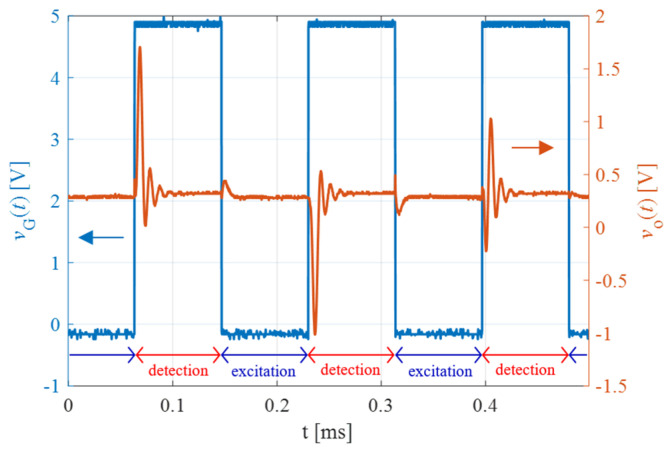
Acquired gating signal *v*_G_(*t*) (blue curve) and demodulated free decaying response *v*_O_(*t*) of the resonator (orange curve) as a function of time.

The contactless reading of the patch sensor was tested within the temperature range 32–46 °C. The IU, shown in Figure 5b, was set up to excite the resonator close to *f*_s_ with *f*_e_ = *ω*_e_/2π = 1.634 MHz and *f*_R_ = *ω*_R_/2π = 1.734 MHz. Figure 6 shows the measured gating signal *v*_G_(*t*) and the output signal *v*_O_(*t*), sampled at 2 MS/s.

**Figure 7:**
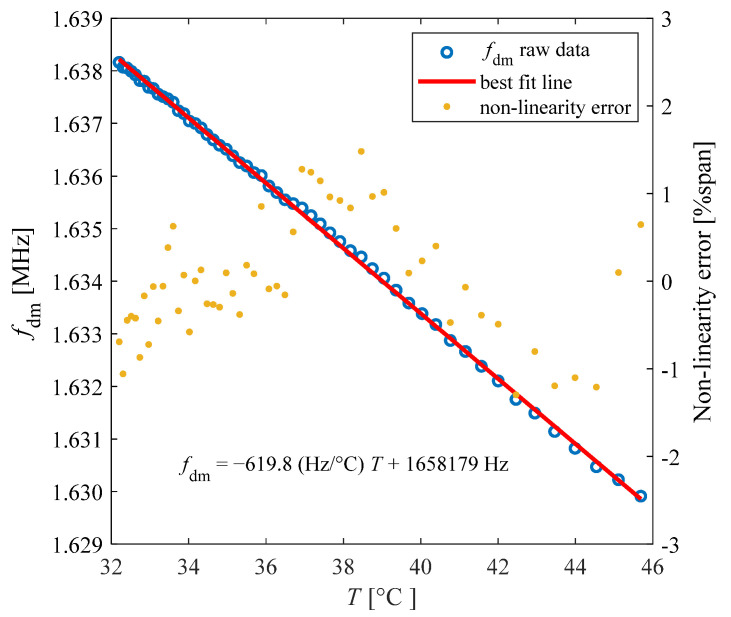
Contactless measurement results of the SU frequency *f*_dm_ (blue circles), best fit line (red line) and non-linearity error (yellow dots) as a function of temperature.

The frequency *f*_dm_ = *ω*_dm_/2π was derived via the autocorrelation function as expressed in (3). Figure 7 shows the obtained values of *f*_dm_, measured as a function of temperature *T*.

The best-fit line for the experimental data provides a temperature sensitivity *S* of −619.8 Hz/°C, with a non-linearity error within ±1.48% of the span of about 8.3 kHz for the explored temperature range.

**Figure 8:**
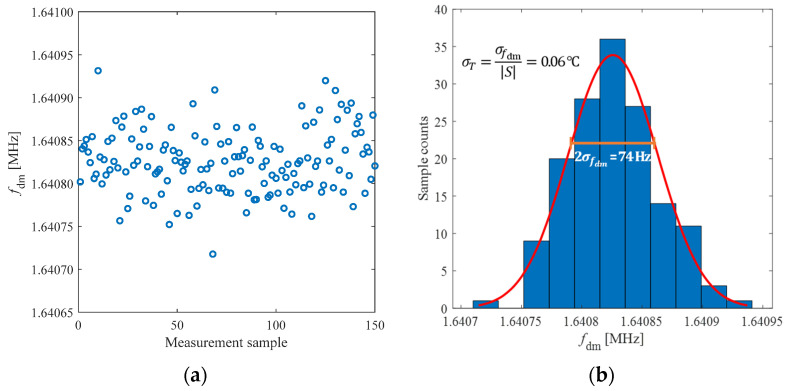
(**a**) Results of 150 repeated measurements of *f*_dm_ at a constant temperature of 28 °C; (**b**) distribution histogram of the 150 measurement results with bins of 214 Hz.

At the constant temperature of 28 °C, 150 repeated measurements of the frequency *f*_dm_ were performed, obtaining the results plotted in Figure 8a. The distribution histogram shown in Figure 8b leads to a standard deviation for *f*_dm_ of *σ_f_*_dm_ = 37 Hz. Considering the sensitivity *S*, the corresponding standard deviation for the temperature *σ_T_* = *σ_f_*_dm_/|*S*| results equal 0.06 °C, which can be considered the equivalent temperature resolution at one *σ*. This value is compliant with the application for body-temperature measurements. Considering the maximum variation Δ*f_s_* due to the variation of *L*_2_ caused by the bending of the patch for a minimum curvature radius of 73 mm, the corresponding maximum error in the temperature reading is Δ*f_s_*/*S* = 0.019 °C, which is lower than the obtained resolution. Since the frequency *f*_s_ is mainly affected by *L*_3_, and since *L*_3_ is not affected by bending, the temperature measurements achieved at different curvature radii provide comparable results.

**Figure 9:**
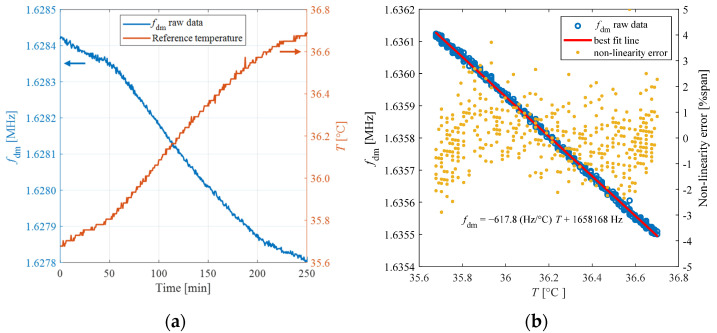
(**a**) Reference temperature *T* measured with a Pt1000 sensor (orange curve) and *f*_dm_ (blue curve) as a function of time; (**b**) *f*_dm_ (blue circles), best fit line (red line) and non-linearity error (yellow dots) as a function of *T* measured versus time.

The stability of the proposed system over time was validated by placing the sensor patch in the testing chamber of Figure 5a at a temperature of about 36 °C while reading a reference temperature with a Pt1000 sensor and the resonant frequency of the patch every 1 min over a timeframe of 4 h. Figure 9a shows the reference temperature *T* and *f*_dm_ as a function of time, while Figure 9b shows the values of *f*_dm_ as a function of *T*.

The best-fit line for the experimental data gives a temperature sensitivity S of −617.8 Hz/°C, in good agreement with the sensitivity obtained exploring the temperature range 32–46 °C. The obtained confirmed relationship between resonant frequency and temperature proves the absence of any significant drift in the sensor output on a time scale of hours.

## 4. Conclusions

A passive flexible patch for body-temperature measurements combining contact sensing with contactless readout by a nearby interrogation unit has been presented. The flexible patch, forming the sensor unit, is composed of an inductive copper coil for magnetic coupling, a ceramic capacitor as the temperature-sensing element based on its TCC, and an additional inductor to make the resonant frequency of the resulting resonant RLC circuit independent of the bending of the patch. The contactless reading exploits the magnetic coupling between the interrogation and sensor units and operates by switching between excitation and detection phases. The readout signal is down-mixed with a reference signal and the frequency of the sensor unit related to the measured temperature is obtained by a digital elaboration based on autocorrelation. A proof-of-concept prototype was developed by employing a paper-based flexible patch and off-the-shelf components. The prototype was experimentally tested within the temperature range 32–46 °C, offering a sensitivity of −619.8 Hz/°C and a resolution of 0.06 °C. Thanks to the additional inductor introduced in the resonant RLC circuit of the patch, the maximum variation of the resonant frequency due to the effect of the patch-bending was reduced to 7.5 ppm for a minimum curvature radius of 73 mm, leading to an equivalent maximum error in the temperature reading of 0.019 °C.

Future developments will consider the fabrication of the patch sensor by adopting a biocompatible substrate to allow for clinical experimentation.

## Data Availability

The data presented in this study are available on request from the corresponding author.
